# Long-term Clinical Outcomes in Favorable Risk Prostate Cancer Patients Receiving Proton Beam Therapy

**DOI:** 10.14338/IJPT-21-00016

**Published:** 2021-10-20

**Authors:** Alicia Bao, Andrew R. Barsky, Russell Maxwell, Justin E. Bekelman, Stefan Both, John P. Christodouleas, Curtiland Deville, Penny Fang, Zelig A. Tochner, Neha Vapiwala

**Affiliations:** 1 Ohio State College of Medicine, The Ohio State University, Columbus, OH, USA; 2 Department of Radiation Oncology, University of Pennsylvania, Philadelphia, PA, USA; 3 Department of Radiation Oncology, University Medical Center Groningen, Groningen, the Netherlands; 4 Department of Radiation Oncology and Molecular Radiation Sciences, Johns Hopkins University, Baltimore, MD, USA; 5 Department of Radiation Oncology, MD Anderson Cancer Center, Houston, TX, USA

**Keywords:** prostate cancer, radiotherapy, proton beam therapy

## Abstract

**Purpose:**

Long-term data regarding the disease control outcomes of proton beam therapy (PBT) for patients with favorable risk intact prostate cancer (PC) are limited. Herein, we report our institution's long-term disease control outcomes in PC patients with clinically localized disease who received PBT as primary treatment.

**Methods:**

One hundred sixty-six favorable risk PC patients who received definitive PBT to the prostate gland at our institution from 2010 to 2012 were retrospectively assessed. The outcomes studied were biochemical failure-free survival (BFFS), biochemical failure, local failure, regional failure, distant failure, PC-specific survival, and overall survival. Patterns of failure were also analyzed. Multivariate Cox proportional hazards modeling was used to estimate independent predictors of BFFS.

**Results:**

The median length of follow-up was 8.3 years (range, 1.2–10.5 years). The majority of patients had low-risk disease (58%, n = 96), with a median age of 64 years at the onset of treatment. Of 166 treated men, 13 (7.8%), 8 (4.8%), 2 (1.2%) patient(s) experienced biochemical failure, local failure, regional failure, respectively. Regional failure was seen in an obturator lymph node in 1 patient and the external iliac lymph nodes in the other. None of the patients experienced distant failure. There were 5 (3.0%) deaths, none of which were due to PC. The 5- and 8-year BFFS rate were 97% and 92%, respectively. None of the clinical disease characteristics or treatment-related factors assessed were associated with BFFS on multivariate Cox proportional hazards modeling (all *P >* .05).

**Conclusion:**

Disease control rates reported in our assessment of PBT were similar to those reported in previous clinically localized intact PC analyses, which used intensity-modulated radiotherapy, three-dimensional conformal radiotherapy, or radical prostatectomy as definitive therapy. In addition, BFFS rates were similar, if not improved, to previous PBT studies.

## INTRODUCTION

Despite the controversy and subsequent changes in prostate-specific antigen (PSA) screening recommendations, prostate cancer (PC) remains the most common nonskin cancer diagnosis for males in the United States [1]. Approximately 40% of newly diagnosed patients present with low-risk disease, which is defined as grade group 1, PSA less than 10 ng/mL, and clinical stage T1c to T2a [2, 3]. A subset of these patients can be further stratified into very-low risk disease, which the National Comprehensive Cancer Network defines as having clinical stage T1c, grade group 1, PSA less than 10 ng/mL, fewer than 3 prostate biopsy fragments/cores positive, less than 50% cancer in each fragment/core, and PSA density less than 0.15 ng/mL/kg [4]. A growing body of evidence supports active surveillance (AS) as the preferred treatment option for patients with very low-risk (VLR) and low-risk (LR) disease, with the benefit of avoiding treatment-related side effects for PC that is unlikely to progress. Although still an option for favorable intermediate risk (FIR) patients, AS is not routinely recommended for all patients in this cohort, as it may confer a higher rate of metastatic progression [5]. Furthermore, there remains a group of patients who do not elect or are not ideal, reliable candidates for AS. Other established treatment options for favorable risk PC include radical prostatectomy (RP) and brachytherapy, but a significant portion of patients are not clinically suitable, or wish to avoid, these invasive procedures.

Evidence-based guidelines promote the use of external-beam (EB) radiotherapy (RT) as an equally effective and appealing noninvasive option for primary, adjuvant, or salvage therapy for PC disease control. Long-term outcomes from the ProtecT randomized, controlled trial show that RT and RP both decrease the risk of disease progression and distant metastasis compared with AS in patients with localized PC [6]. Therefore, RT continues to be an efficacious therapy choice for patients who do not opt for or are ineligible for AS or surgery.

EBRT has undergone numerous technologic advancements to more precisely target the tumor, while sparing nearby organs. The current standard of care EBRT technique for treating PC is intensity-modulated RT (IMRT) [7, 8]. Proton beam therapy (PBT) is a relatively newer form that is becoming increasingly available and applied. The unique, physical characteristics of PBT may offer dosimetric advantages to nearby organs at risk, potentially decreasing the likelihood of gastrointestinal or genitourinary adverse effects [9]. These toxicity studies have been thoroughly conducted. Several studies have reported similar adverse event rates and quality-of-life outcomes among PC patients treated with PBT versus IMRT [10, 11]. In addition, multiple dosimetric modeling systems suggest that PBT may be superior to photon RT in reducing the likelihood of secondary malignancies [12].

Although PBT has been used to treat PC for many years, there is a relative scarcity of published long-term data on disease control outcomes and patterns of failure with more modern PBT techniques. Previous studies have demonstrated reasonable disease control using combination photon-proton therapy, or PBT alone with lower dose constraints [13, 14]. However, long-term disease control outcome data following high-dose PBT in PC patients with clinically localized disease remain limited. In this analysis, we report long-term disease control outcomes and patterns of failure in patients with favorable risk disease receiving primary PBT for PC control.

## METHODS

### Patient Selection

We conducted an institutional review board approved retrospective analysis of 166 PC patients with favorable risk disease (VLR, LR, and FIR) who received PBT between January 2010 and December 2012. The patient population in this study has been previously described in another publication, and are also currently enrolled in a prospective study on long-term toxicity outcomes [10]. Patients treated after 2012 were not included to compare 8-year survival results with previously existing literature [13,15]. All patients had histologically confirmed prostate adenocarcinoma, and any patients with metastatic disease or pelvic lymph node (LN) involvement at diagnosis were excluded. All patients received PBT as their sole RT modality.

### Treatment Delivery

All patients were simulated and treated with endorectal balloons, using image-guided RT, optimization, and planning methods as previously described [10]. The clinical target volume was defined as the whole prostate plus 1 cm of the proximal seminal vesicles [16], and the planning target volume being defined as a 0.5-cm uniform expansion from the clinical target volume. Specific dosimetric parameters to the target volumes, as well as to the bladder and rectum, can be found in **Supplemental Table 1**. Patients were placed in a supine position and treated with 2 parallel opposed fields (90° and 270°). A range correction was added in the direction of each beam to ensure treatment robustness. Lateral margins were increased up to 1 cm during the IMPT optimization process. All patients were prescribed 79.2 Gy (relative biological effectiveness [RBE] = 1.1) in 44 fractions to the clinical target volume. Elective nodal radiation was not delivered.

### Clinical Assessment

The primary outcome measure in this study was biochemical failure–free survival (BFFS) based on initial risk groups in patients undergoing PBT for favorable risk PC. Patients were retrospectively assessed for baseline characteristics and clinical endpoints by the first author and confirmed by the second and senior authors. Serial measurements of PSA were obtained every 3 months for the first 5 years after treatment, then every 6 months thereafter. BF was described according to the Phoenix definition as a rise in prostate-specific antigen (PSA) of 2 ng/mL or greater above the patient's post-RT nadir [17]. Biochemical failure was followed up with pelvic computed tomography, prostate magnetic resonance imaging, and/or technetium bone scan, guided by clinical evidence and the patient's PSA levels. When feasible and if deemed to potentially change clinical management, a prostate biopsy procedure was pursued to confirm recurrence. Local failure (LF) within the prostate or seminal vesicles was described by a recurrence confirmed by either conventional imaging (computed tomography or magnetic resonance imaging) and/or prostatic biopsy procedure. Regional failure (RF) was used to describe metastasis to pelvic LN, and distant failure (DF) was used to describe all other distant nodal, bony, or solid organ metastases. PC-specific survival (PCSS) and overall survival (OS) were also reported. All survival metrics were calculated from start of RT.

### Statistical Assessment

The Kaplan-Meier (KM) method was used to generated BFFS, LF, RF, DF, PCSS, and OS curves. Cox univariate and multivariate analyses were conducted using SAS version 9.2. KM estimates and plots were created using GraphPad Prism 8 (GraphPad Software, San Diego, CA). All analyses were considered statistically significant if 2-tailed *P* values were less than a type 1 error rate set at .05.

## RESULTS

**Figure 1.**
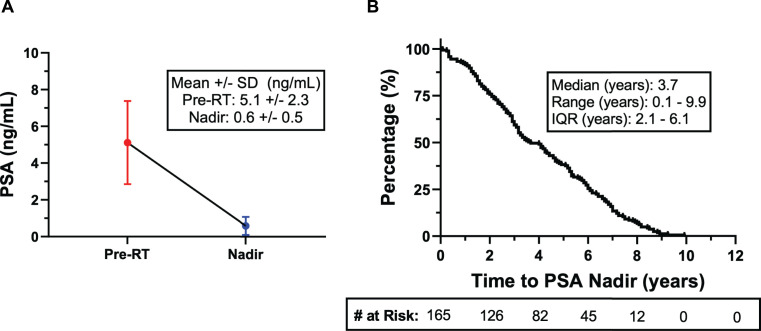
(A) Mean PSA at start of RT and mean PSA nadir after RT. (B) Median time to PSA nadir. Abbreviations: PSA, prostate-specific antigen; RT, radiotherapy; SD, standard deviation; IQR, interquartile range.

Baseline cohort characteristics are described in **Table 1**. The median age at the start of RT was 64 years (range, 42–82 years). Per the institutional review board–approved protocol and typical fractionation that were in effect during that time, all patients were prescribed a treatment dose of 79.2 GyRBE in 44 fractions. One hundred twenty patients (72%) received double-scattering technique and 46 patients (28%) received non-double scattering technique, as it became available at our institution. Patients in our cohort had VLR (n = 45, 27%), LR (n = 96, 58%), or FIR (n = 25, 15%) [4]. The mean PSA at diagnosis was 5.1 ng/mL ±2.3 ng/mL and the mean PSA nadir after RT was 0.6 ng/mL ±0.5 ng/mL (**Figure 1A**). The PSA nadir by risk group was 0.6, 0.5, and 0.4 ng/mL for the VLR, LR, and FIR groups, respectively. The overall median time to PSA nadir after RT was 3.7 years (**Figure 1B**). Median times to PSA nadir were 4.5, 3.2, and 4.4 years for the VLR, LR, and FIR groups, respectively. Two patients (1.2%) with FIR disease received concurrent ADT, with a median length of ADT of 6.5 months. The median follow-up at the time of analysis was 8.3 years (range, 1.2–10.5 years).

**Table 1.** Proton therapy cohort baseline demographic, clinical, and treatment characteristics.

**Variable**	**Value**
N	166
Age, median (range, IQR), y	64 (42–82, 59–69)
Race, n (%)	
White	136 (82)
Non-white	30 (18)
Risk group, n (%)	
Very low	45 (27)
Low	96 (58)
Favorable intermediate	25 (15)
Pre-RT PSA, mean ± SD (range, IQR) ng/mL	5.1 ± 2.3 (0.3–14, 3.6–6.4)
Proton modality, n (%)	
Double scattering	120 (72)
Non-double scattering	46 (28)
Concurrent ADT, n (%)	
Yes	2 (1)
No	164 (99)
Pre-RT ECOG performance status, n (%)	
0	161 (97)
1	5 (3)

Abbreviations: IQR, interquartile range; RT, radiotherapy; PSA, prostate-specific antigen; SD, standard deviation; ADT, androgen deprivation therapy; ECOG, Eastern Cooperative Oncology Group.

Percentages will not always total 100% due to rounding of decimals.

**Figure 2.**
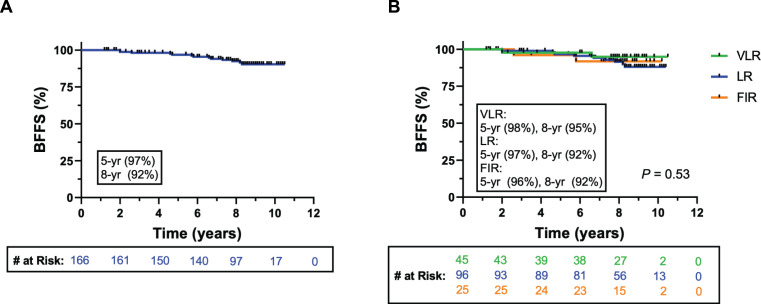
(A) Kaplan-Meier estimates of BFFS for intact prostate cancer patients receiving PBT. (B) BFFS stratified by NCCN risk groups. Abbreviations: BFFS, biochemical failure-free survival; NCCN, National Comprehensive Cancer Network; PBT, proton beam therapy; VLR, very low-risk; LR, low-risk; FIR, favorable intermediate-risk.

**Figure 3.**
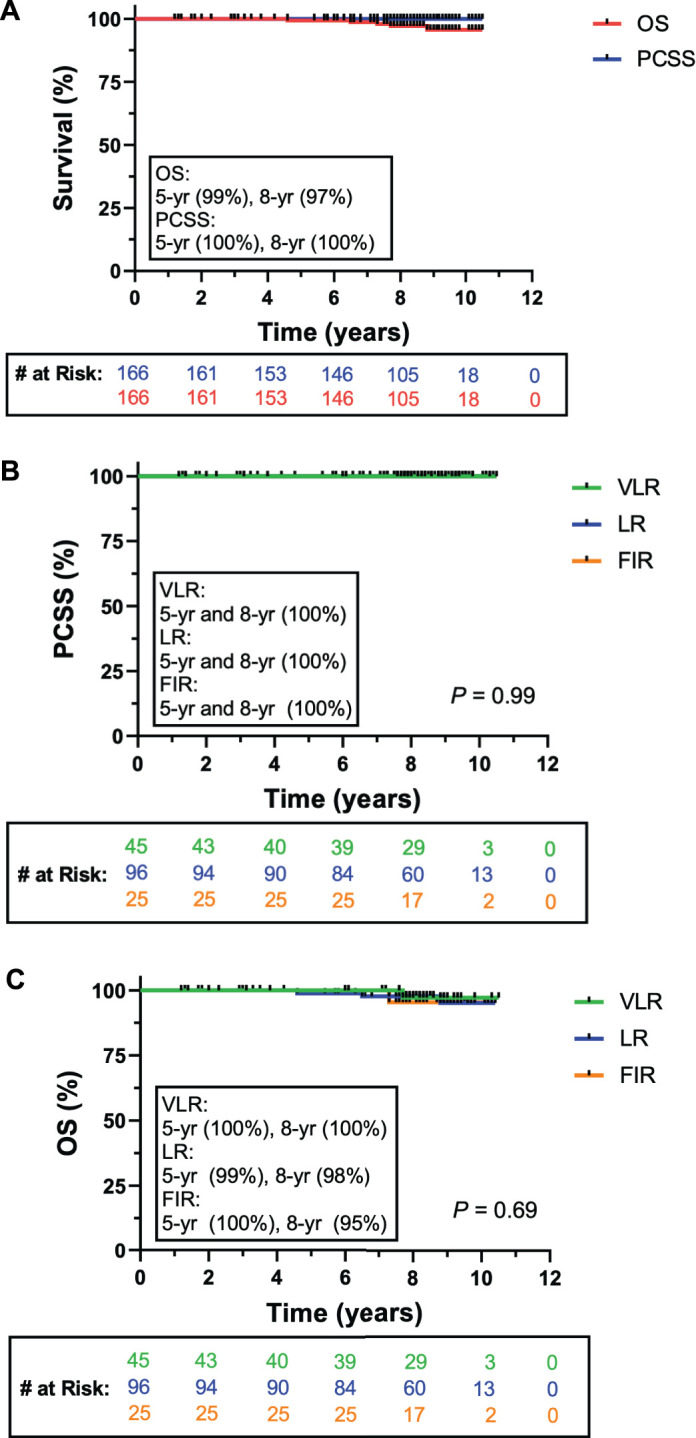
(A) Kaplan-Meier estimates of overall survival (red) and PCSS (blue) for intact PC patients receiving PBT. (B) PCSS stratified by NCCN risk group. (C) OS stratified by NCCN risk group. Abbreviations: NCCN, National Comprehensive Cancer Network; OS, overall survival; PBT, proton beam therapy; PC, prostate cancer; PCSS, prostate cancer–specific survival; VLR, very low-risk; LR, low-risk; FIR, favorable intermediate-risk.

Patterns of failure are reported in **Table 2**. BF occurred in 13 patients (7.8%) at a median of 5.8 years (range, 2.0–8.3 years) from start of RT. Of BF events, 2 (4.4%) occurred in the VLR group, 9 (9.3%) occurred in the LR group, and 2 (8.0%) in the FIR group. Of patients who experienced BF, LF within the prostate was found in 8 patients (4.8%) at a median of 6.3 years (range, 3.0–10.1 years) from start of RT. In all 8 patients, LF was initially detected in the treated prostate by magnetic resonance imaging, and subsequently confirmed in 7 patients by needle biopsy procedure. RF was observed in 2 patients (1.2%), at a median of 6.7 years from the start of RT as follows: 1 in an obturator LN and the other in the external iliac LNs. This was diagnosed using fluciclovine positron-emission tomography/computed tomography. Both cases of RF occurred in patients with LR disease. Of patients with LF, 3 (1.8%) underwent salvage RP and 2 (1.2%) received salvage brachytherapy, at a median of 7.5 years from start of PBT. Two patients experienced another BF postprostatectomy, with 1 progressing to RF in the external iliac LNs. No incidences of DF were noted in this cohort. There were 5 (3.0%) deaths in this cohort over the study period, but none were attributable to PC. The KM estimates for BFFS in the overall cohort and divided by risk group are shown in **Figure 2**. The 5- and 8-year BFFS rate were 97% (95% CI 95%–100%) and 92% (95% CI 89%–98%), respectively (**Figure 2A**). The 5- and 8-year BFFS divided by risk group was 98% (95% CI 96%–100%) and 95% (95% CI 91%–100%) for the VLR group, 97% (95% CI 94%–100%) and 92% (95% CI 87%–100%) for the LR group, and 96% (95% CI 93%–100%) and 92% (95% CI 85%–100%) in the FIR group (**Figure 2B**). The 5- and 8-year OS for the entire cohort was 99% and 97%, respectively (**Figure 3A**). PCSS was 100% across all risk groups, as no patient deaths were attributable to PC (**Figure 3A, 3B**). Eight-year OS was 100%, 98%, and 95% in the VLR, LR, and FIR groups, respectively (**Figure 3B**).

**Table 2.** Patterns of failure analysis.

**Clinical Outcome**	**Patients, n/total (%)**	**Sites of failure (n/total, %)**
Biochemical failure	13/166 (8)	N/A
Local failure, prostate	9/166 (5)	9/9 (100)
Regional failure	2/166 (1)	
External iliac LN		1/2 (50)
Obturator LN		1/2 (50)
Distant failure	0/166 (0)	N/A
PC-specific mortality	0/166 (0)	N/A
All-cause mortality	5/166 (3)	N/A

Abbreviations: LN, lymph node; N/A, not applicable; PC, prostate cancer.

A Cox regression was used to identify predictors of BFFS, such as age, race, risk group, pre-RT PSA, concurrent ADT, and PBT modality. On univariate and multivariate analyses, none of the clinical disease characteristics or treatment related factors studied were associated with BFFS (all *P* > .05) (**Table 3**).

**Table 3.** Univariate and multivariate cox regression analyses of predictors of BFFS.

**Characteristic**	**UVA**		**MVA**	
	**HR (95% CI)**	***P*** **value^a^**	**HR (95% CI)**	***P*** **value**
Age at diagnosis, years (unit = 10 y)	0.89 (0.42–1.19)	.77	0.86 (0.40–1.88)	.71
Race				
White	REF		REF	
Non-white	1.27 (0.35–4.63)	.71	1.24 (0.33–4.58)	.75
Pre-RT PSA, ng/mL (unit = 5 ng/mL)	1.31 (0.42–4.08)	.64	1.25 (0.40–3.85)	.70
Risk group				
Very low	REF		REF	
Low	2.04 (0.44–9.45)	.36	1.98 (0.43–9.20)	.34
Favorable intermediate	1.71 (0.24–12.15)	.59	1.92 (0.26–14.11)	.52
Concurrent ADT				
No	REF		REF	
Yes	<0.1 (undefined)	.99	<0.1 (undefined)	.99
Proton modality				
DS	REF		REF	
Non-DS	1.68 (0.55–5.15)	.36	1.72 (0.56–5.30)	.34
Pre-RT ECOG performance status				
0	REF		REF	
1	<0.1 (undefined)	.99	<0.1 (undefined)	.99

Abbreviations: BFFS, biochemical failure-free survival; UVA, univariate analysis; MVA, multivariate analysis; HR. hazard ratio; REF, reference; RT, radiotherapy; PSA, prostate-specific antigen; ADT, androgen-deprivation therapy; DS, double-scattering; ECOG, Eastern Cooperative Oncology Group.

a All tests were 2-tailed, and statistical significance was set at a threshold *P* < .05.

## DISCUSSION

In this report, we provide long-term disease control and patterns of failure results for the use of dose-escalated (79.2 GyRBE) PBT with relatively modern techniques in the treatment of intact PC for a low- and intermediate-risk cohort, offering a unique analysis evaluating 5 or more year outcomes following dose-escalated PBT in the intact PC population. At a median follow-up of 8.3 years from start of PBT, we found BF, LF, RF, and DF rates of 7.8%, 4.8%, 1.1%, and 0.0%, respectively. KM analysis showed favorable survival outcomes, with 5- and 8-year BFFS of 97% (95% CI 95%–100%) and 92% (95% CI 89%–98%), respectively, for the entire cohort. As expected, the survival outcomes were improved in the VLR cohort (5- and 8-year BFFS of 98% [95% CI 96%–100%] and 95% [95% CI 91%–100%], respectively) compared with the LR (5- and 8-year BFFS of 97% [95% CI 94%–100%] and 92% [95% CI 87%-100%], respectively) and FIR (5- and 8-year BFFS of 96% [95% CI 93%–100%] and 92% [95% CI 85%–100%] cohort, respectively). Of 8 patients who experienced LF, 5 underwent salvage therapies. Two patients who underwent salvage RP experienced a second BF. Even among the patients in our study who ultimately experienced recurrences after their initial treatment, PBT alone provided a median of 7.5 progression-free years before salvage therapy was initiated.

The BF rate observed in our cohort, 9% at a median of 8.3 years from start of PBT, aligns well with the failure rates reported in previous photon RT studies [6,15,19]. Given its design, the ProtecT trial represents the highest level of evidence among studies comparing PC control following RP, RT, or AS in a primarily low-risk cohort (78%) [6]. The ProtecT study found a BF rate of 14% in their RT arm, which consisted of three-dimensional conformal RT (3DCRT) delivered to a dose of 74 GyRBE in 37 fractions, based on the guidelines set forth in the RTO1 trial [19, 20].

However, since enrollment in the ProtecT study ended, new evidence has suggested that dose-escalated RT improves BFFS in both low- and intermediate-risk patients. The 2010 PROG 95-09 trial was conducted with a fixed 3D-CRT dose (50.4 GyRBE) boosted with either a conventional dose (70.2 GyRBE) or escalated dose (79.2 GyRBE) of protons [14]. The dose-escalated arm exhibited a significantly lower 10-year BF rate than the conventional dose arm (*P* < .001). In addition, the more recent RTOG 0126 study released 10-year outcomes after a comparison of conventional dose (70.2 GyRBE) and dose-escalated (79.2 GyRBE) 3DCRT or IMRT, which showed significantly lower BF rates in the high-dose arm (*P* < .001) [15]. The PROG 95-09 trial reported a 10-year BF rate of 17.4% in their low-risk cohort treated with the escalated 79.2 GyRBE dose, and then RTOG 0126 reported a 5-year BF rate of 13% in their 79.2 GyRBE arm [14,15]. Although it is difficult to make direct comparisons between our data and these prospective studies, our data demonstrate that 79.2 GyRBE delivered using PBT provides a reassuring level of biochemical control comparable to, if not better than, the same dose delivered via photon-based approaches.

One of the earliest PBT studies was conducted at Loma Linda University, which provided initial support for the use of lower dose (74 GyRBE) PBT in PC treatment [13]. Unlike our study, their results reflected a mixed cohort in which some patients received combination photon-proton therapy and some who received PBT only. From this study, they reported 5- and 8-year BFFS of 75% and 73%, respectively. The clinical outcomes presented in this paper closely align with the results published in a large-scale retrospective PBT study done by Takagi et al [20], where majority of the study patients were treated using the same dose (74 GyRBE) and fractionation protocol as the ProtecT trial. Their study demonstrated favorable survival across all risk groups, with a 100% (95% CI, 100–100), 98.5% (95% CI, 96.0–99.4), and 93% (95% CI, 89.4–95.4) 5-year BFFS in their VLR, LR, and FIR cohorts, respectively [21]. We report similar long-term BFFS results of 98% (95% CI 96%–100%), 97% (95% CI 94%–100%), and 96% (95% CI 93%–100%) in the VLR, LR, and IFR risk groups, respectively, using a dose escalated 79.2 GyRBE. Notably, the Takagi et al [20] paper described a higher incidence of BF in their younger patients, particularly those older than 64 years. While unable to be directly compared, our VLR (median 63 years) and LR (median 64 years) patients showed similar BFFS outcomes to the entire Takagi cohort [20], despite being younger on average. Another retrospective study similar to ours was conducted at the University of Florida, where they reported a similar 5-year BFFS of 99% in their low-risk PC patients, but using dose-escalated 78 GyRBE PBT [22]. A direct comparison of the 5-year BFFS results reported in this study to other RT studies conducted in favorable risk prostate cancer patients can be found in **Supplemental Table 2**.

In comparing the use of RT to other treatment options, the results of the ProtecT trial reported no significant differences in progression-free survival between their RT (5174 person-years) and RP (5138 person-years) arms [6]. The ProtecT results are echoed by 10-year follow-up data from a newly published, retrospective analysis of 1503 intermediate-risk PC patients. With and without propensity score matching, nearly identical 10-year BFFS was observed between the RT and RP group (unadjusted 58.0% prostatectomy vs 58.5% RT, adjusted 57.1% prostatectomy vs 57.0% RT) [23]. Both of these studies used 3DCRT as the RT modality. To date, no randomized, controlled trial has directly studied the comparison between PBT, RP, or AS. However, our PBT results yielded a favorable 8-year BFFS, thus PBT alone provides a reasonable alternative to RP for PC patients who wish to avoid an invasive surgical procedure.

While we provide a long-term report of BFFS in PC patients with intact disease, a similar single-arm report of PBT in PC patients has already demonstrated reasonable disease control in the postprostatectomy setting [24]. Furthermore, a retrospective, case-matched analysis comparing PBT with IMRT after RP showed no significant differences in BF rates between the 2 study groups, thus supporting comparable efficacy of the 2 modalities in treating PC in the postoperative setting [25]. Our current data, considered in conjunction with the postprostatectomy PBT results, provide further support for the clinical efficacy of PBT as a treatment modality for PC across a variety of settings.

Although gastrointestinal and genitourinary toxicity and RT dose to nearby organs at risk are important considerations in PBT, this analysis was meant to focus on PC disease control. The initial toxicity outcomes in our cohort have previously been published, and analysis of longer-term toxicities is planned for a future study [10]. In the initial report, on multivariate analysis, there were no significant differences in acute or late gastrointestinal or genitourinary toxicities between PBT and IMRT, at a median of 29 months of follow-up in the PBT arm. Taken together, PBT should be considered an effective and safe modality for the treatment of intact PC, with reasonable long-term disease control outcomes reported in this analysis, and an initial toxicity profile comparable to that of IMRT, as shown in the prior analysis.

This study has several important limitations. The data presented herein are retrospective, though all patients were treated and seen at a single, large institution, thereby reducing the likelihood for information bias or variable recordkeeping. Cox regression analysis was used to identify factors that might confound BFFS, but none of the characteristics were found to be significant. One possible limitation was that the cohort was relatively uniform by design, and the relatively low number of intermediate- and high-risk patients may have precluded observation of any statistically significant findings. Another notable limitation is that practice patterns for low-risk prostate cancer patients have evolved since this study was initiated, and data from studies, such as ProtecT and RTOG 0415, have further established the role of AS and moderate hypofractionation, respectively [6,26]. Nonetheless, we believe that the long-term follow-up of our cohort still offers a useful perspective on PC patients with favorable risk disease treated with an accepted standard of care at that time. Our report specifically provides benchmark disease control outcome data on the use of PBT in this population, contributing data to the conversation when counseling patients on their options, and for comparison with our moderately hypofractionated proton radiotherapy experience [27]. Despite representing a considerably large cohort of PBT patients to come out of a single-treatment center, the overall cohort size yields relatively small statistical power. Overall incidences of LF, RF, and DF events were relatively, and not surprisingly, low; extended follow-up time with a larger cohort may reveal further clinically meaningful findings in BFFS.

This report provides supportive evidence of the efficacy of PBT as an initial treatment for low- to intermediate-risk PC. Future directions of study will include a case-matched, comparative analysis of long-term outcomes with PBT versus IMRT for patients with PC with intact disease, and a review of late toxicities to update our initial toxicity report for this cohort. As we eagerly await prospective, randomized data from the PARTIQoL (NCT01617161) and COMPPARE (NCT03561220) trials for more definitive guidance on modality-based outcome differences, attempts to help build the evidence base continue [28, 29]. A recent multicenter pooled analysis has evaluated toxicity differences between moderately fractionated PBT and IMRT [30]. Our analysis now offers further support that with continued close follow-up, PBT remains a safe and effective RT option for treating intact PC in patients with long expected survivals, and thus long periods for manifesting both toxicity and disease recurrence.

### Conclusions

Our study represents a long-term analysis of BFFS, survival outcomes, and patterns of failure in PC patients treated with PBT in a cohort with clinically localized disease. Comparisons to photon RT studies demonstrate that even with a predominantly double-scattering technique, PBT offers similar PC disease control to modern photon therapies. Considered in the context of other data demonstrating relatively low toxicity outcomes of PBT in both the intact and postprostatectomy setting, this report further complements what is now a solid and growing evidence base for the long-term safety and efficacy of PBT for PC patients.

## References

[1] Siegel RL, Miller KD, Jemal A (2019). Cancer statistics, 2019. *CA Cancer J Clin*.

[2] D'Amico AV, Whittington R, Malkowicz SB, Cote K, Loffredo M, Schultz D, Chen MH, Tomaszewski JE, Renshaw AA, Wein A, Richie JP (2002). Biochemical outcome after radical prostatectomy or external beam radiation therapy for patients with clinically localized prostate carcinoma in the prostate specific antigen era. *Cancer*.

[3] Jang TL, Yossepowitch O, Bianco FJ, Scardino PT (2007). Low risk prostate cancer in men under age 65: The case for definitive treatment. *Urol Oncol Semin Orig Investig*.

[4] Freedman-Cass D, Shead DA, Schaeffer E, Antonarakis ES, Armstrong AJ, Bekelman JE, Cheng H, Victor AD, Davis BJ, Desai N, Dorff T, Eastham JA, Farrington TA, Gao X, Mark Horwitz E, Ippolito JE, Kuettel MR, Lang JM, McKay R, McKenney J, Netto G, Penson DF, Pow-Sang JM, Reiter R, Richey S, Jude S, Roach M, Rosenfeld S, Shabsigh A, Spratt DE, Teply BA, Tward J NCCN guidelines version 2.2021 prostate cancer; 2021. National Comprehensive Cancer Network Accessed Mo D Yr.

[5] Klotz L (2017). Active Surveillance for Intermediate Risk Prostate Cancer. *Curr Urol Rep*.

[6] Hamdy FC, Donovan JL, Lane JA, Mason M, Metcalfe C, Holding P, Davis M, Peters TJ, Turner EL, Martin RM, Oxley J, Robinson M, Staffurth J, Walsh E, Bollina P, Catto J, Doble A, Doherty A, Gillatt D, Kockelbergh R, Kynaston H, Paul A, Powell P, Prescott S, Rosario DJ, Rowe E, Neal DE (2016). 10-year outcomes after monitoring, surgery, or radiotherapy for localized prostate cancer. *N Engl J Med*.

[7] Viani G, Hamamura AC, Faustino AC (2019). Intensity modulated radiotherapy (IMRT) or conformational radiotherapy (3D-CRT) with conventional fractionation for prostate cancer: Is there any clinical difference?. *Int Braz J Urol*.

[8] Viani GA, Viana BS, Martin JEC, Rossi BT, Zuliani G, Stefano EJ (2016). Intensity-modulated radiotherapy reduces toxicity with similar biochemical control compared with 3-dimensional conformal radiotherapy for prostate cancer: a randomized clinical trial. *Cancer*.

[9] Vargas C, Fryer A, Mahajan C, Indelicato D, Horne D, Chellini A, McKenzie C, Lawlor P, Henderson R, Li Z, Lin L, Olivier K, Keole S (2008). Dose-volume comparison of proton therapy and intensity-modulated radiotherapy for prostate cancer. *Int J Radiat Oncol Biol Phys*.

[10] Fang P, Mick R, Deville C, Both S, Bekelman JE, Christodouleas JP, Guzzo TJ, Tochner Z, Hahn SM, Vapiwala N (2015). A case-matched study of toxicity outcomes after proton therapy and intensity-modulated radiation therapy for prostate cancer. *Cancer*.

[11] Hoppe BS, Michalski JM, Mendenhall NP, Morris CG, Henderson RH, Nichols RC, Mendenhall WM, Williams CR, Regan MM, Chipman JJ, Crociani CM, Sandler HM, Sanda MG, Hamstra DA (2014). Comparative effectiveness study of patient-reported outcomes after proton therapy or intensity-modulated radiotherapy for prostate cancer. *Cancer*.

[12] Eaton BR, MacDonald SM, Yock TI, Tarbell NJ (2015). Secondary malignancy risk following proton radiation therapy. *Front Oncol*.

[13] Slater JD, Rossi CJ, Yonemoto LT, Bush DA, Jabola BR, Levy RP, Grove RI, Preston W, Slater JM (2004). Proton therapy for prostate cancer: the initial Loma Linda University experience. *Int J Radiat Oncol Biol Phys*.

[14] Zietman AL, Bae K, Slater JD, Shipley WU, Efstathiou JA, Coen JJ, Bush DA, Lunt M, Spiegel DY, Skowronski R, Jabola BR, Rossi CJ (2010). Randomized trial comparing conventional-dose with high-dose conformal radiation therapy in early-stage adenocarcinoma of the prostate: long-term results from Proton Radiation Oncology Group/American College Of Radiology 95-09. *J Clin Oncol*.

[15] Michalski JM, Moughan J, Purdy J, Bosch W, Bruner DW, Bahary JP, Lau H, Duclos M, Parliament M, Morton G, Hamstra D, Seider M, Lock MI, Patel M, Gay H, Vigneault E, Winter K, Sandler H (2018). Effect of standard vs dose-escalated radiation therapy for patients with intermediate-risk prostate cancer the nrg oncology rtog 0126 randomized clinical trial. *JAMA Oncol*.

[16] Meyer J, Bluett J, Amos R, Levy L, Choi S, Nguyen QN, Zhu XR, Gillin M, Lee A (2010). Spot scanning proton beam therapy for prostate cancer: treatment planning technique and analysis of consequences of rotational and translational alignment errors. *Int J Radiat Oncol Biol Phys*.

[17] Abramowitz MC, Li T, Buyyounouski MK, Ross E, Uzzo RG, Pollack A, Horwitz EM (2008). The phoenix definition of biochemical failure predicts for overall survival in patients with prostate cancer. *Cancer*.

[18] Dearnaley DP, Sydes MR, Graham JD, Aird EG, Bottomley D, Cowan RA, Huddart RA, Jose CC, Matthews JH, Millar J, Moore AR, Morgan RC, Russell JM, Scrase CD, Stephens RJ, Syndikus I, Parmar MK; (2007). RT01 collaborators. Escalated-dose versus standard-dose conformal radiotherapy in prostate cancer: first results from the MRC RT01 randomised controlled trial. *Lancet Oncol*.

[19] Lane JA, Donovan JL, Davis M, Walsh E, Dedman D, Down L, Turner EL, Mason MD, Metcalfe C, Peters TJ, Martin RM, Neal DE, Hamdy FC (2014). Active monitoring, radical prostatectomy, or radiotherapy for localised prostate cancer: Study design and diagnostic and baseline results of the ProtecT randomised phase 3 trial. *Lancet Oncol*.

[20] Takagi M, Demizu Y, Fujii O, Terashima K, Niwa Y, Daimon T, Tokumaru S, Fuwa N, Hareyama M, Okimoto T (2021). Proton therapy for localized prostate cancer: long-term results from a single-center experience. *Int J Radiat Oncol Biol Phys*.

[21] Bryant C, Smith TL, Henderson RH, Hoppe BS, Mendenhall WM, Nichols RC, Morris CG, Williams CR, Su Z, Li Z, Lee D, Mendenhall NP (2016). Five-year biochemical results, toxicity, and patient-reported quality of life after delivery of dose-escalated image guided proton therapy for prostate cancer. *Int J Radiat Oncol Biol Phys*.

[22] Goy BW, Burchette R, Soper MS, Chang T, Cosmatos HA (2020). Ten-year treatment outcomes of radical prostatectomy vs external beam radiation therapy vs brachytherapy for 1503 patients with intermediate-risk prostate cancer. *Urology*.

[23] Deville C, Hwang WT, Barsky AR, Both S, Christodouleas JP, Bekelman JE, Tochner Z, Vapiwala N (2020). Initial clinical outcomes for prostate cancer patients undergoing adjuvant or salvage proton therapy after radical prostatectomy. *Acta Oncol (Madr)*.

[24] Barsky AR, Carmona R, Santos PMG, Verma V, Both S, Bekelman JE, Christodouleas JP, Vapiwala N (2020). Comparative clinical outcomes and patterns of failure of proton-beam therapy versus intensity-modulated radiotherapy (IMRT) for prostate cancer in the postoperative setting. *Pract Radiat Oncol*.

[25] Lee WR, Dignam JJ, Amin MB, Bruner DW, Low D, Swanson GP, Shah AB, D'Souza DP, Michalski JM, Dayes IS, Seaward SA, Hall WA, Nguyen PL, Pisansky TM, Faria SL, Chen Y, Koontz BF, Paulus R, Sandler HM (2016). Randomized phase III noninferiority study comparing two radiotherapy fractionation schedules in patients with low-risk prostate cancer. *J Clin Oncol*.

[26] Grewal AS, Schonewolf C, Min EJ, Chao HH, Both S, Lam S, Mazzoni S, Bekelman J, Christodouleas J, Vapiwala N (2019). Four-year outcomes from a prospective phase ii clinical trial of moderately hypofractionated proton therapy for localized prostate cancer. *Int J Radiat Oncol Biol Phys*.

[27] (2020). Proton therapy vs. IMRT for low or intermediate risk prostate cancer (PARTIQoL). ClinicalTrials.gov.identifier.

[28] (2021). A Prospective Comparative Study of Outcomes With Proton and Photon Radiation in Prostate Cancer (COMPPARE). ClinicalTrials.gov.identifier.

[29] Vapiwala N, Wong JK, Handorf E, Paly J, Grewal A, Tendulkar R, Godfrey D, Carpenter D, Mendenhall NP, Henderson RH, Stish BJ, Vargas C, Salama JK, Davis BJ, Horwitz EM (2021). A pooled toxicity analysis of moderately hypofractionated proton beam therapy and intensity modulated radiation therapy in early-stage prostate cancer patients. *Int J Radiat Oncol Biol Phys*.

