# Supplementary material for: Long-term Clinical Outcomes in Favorable Risk Prostate Cancer Patients Receiving Proton Beam Therapy
Source: Int J Part Ther. 2021 Oct 20;8(4):14–24. doi: 10.14338/IJPT-21-00016 (PMC9009454; doi:10.14338/IJPT-21-00016)
Supplement: Supplementary file 1 [file ijpt-08-04-04.docx]

**Supplementary Table 1a.** Dosimetric parameters for clinical target volume (CTV)

| **CTV** | | **Value** |
| --- | --- | --- |
| V95% (%) | Range | 97.57 ­– 100.00 |
|  | Median | 100.00 |
|  | Mean ± SD | 99.98 ± 0.19 |
|  | IQR | 0.00 |
| V98% (%) | Range | 58.41 – 100.00 |
|  | Median | 99.99 |
|  | Mean ± SD | 99.37 ± 3.42 |
|  | IQR | 0.09 |
| V105% (%) | Range | 0.00 – 8.91 |
|  | Median | 0.00 |
|  | Mean ± SD | 0.12 ± 0.79 |
|  | IQR | 0.00 |

*Abbreviations*: CTV, clinical target volume; V95% (%), the volume of structure receiving 95% of the prescription dose, expressed as a percentage; SD, standard deviation; IQR, interquartile range

**Supplementary Table 1b.** Dosimetric parameters for planning target volume (PTV)

| **PTV** | | **Value** |
| --- | --- | --- |
| V95% (%) | Range | 85.66 – 100.00 |
|  | Median | 99.95 |
|  | Mean ± SD | 99.77 ± 1.15 |
|  | IQR | 0.15 |
| V98% (%) | Range | 55.19 – 99.93 |
|  | Median | 96.48 |
|  | Mean ± SD | 95.59 ± 4.54 |
|  | IQR | 3.16 |
| V105% (%) | Range | 0.00 – 6.91 |
|  | Median | 0.00 |
|  | Mean ± SD | 0.09 ± 0.60 |
|  | IQR | 0.00 |

*Abbreviations*: PTV, planning target volume; V95% (%), the volume of structure receiving 95% of the prescription dose, expressed as a percentage; SD, standard deviation; IQR, interquartile range

**Supplementary Table 1c.** Dosimetric parameters for the bladder

| **Bladder** | | **Value** |
| --- | --- | --- |
| V60 Gy (%) | Range | 2.54 – 34.93 |
|  | Median | 10.29 |
|  | Mean ± SD | 12.18 ± 6.98 |
|  | IQR | 7.19 |
| V70 Gy (%) | Range | 1.42 – 25.06 |
|  | Median | 6.97 |
|  | Mean ± SD | 8.54 ± 5.26 |
|  | IQR | 5.30 |
| V80 Gy (cm^3^) | Range | 0.00 – 77.16 |
|  | Median | 0.04 |
|  | Mean ± SD | 3.45 ± 10.31 |
|  | IQR | 2.09 |

*Abbreviations*: V60 Gy (%), the volume of structure receiving a 60 Grey radiation dose, expressed as a percentage; V 80 Gy (cm^3^), the volume of the structure receiving an 80 Grey radiation dose, in cubic centimeters; SD, standard deviation; IQR, interquartile range

**Supplementary Table 1d.** Dosimetric parameters for the rectum

| **Rectum** | | **Value** |
| --- | --- | --- |
| V40 Gy (%) | Range | 11.15 – 35.86 |
|  | Median | 23.63 |
|  | Mean ± SD | 23.36 ± 4.58 |
|  | IQR | 6.05 |
| V60 Gy (%) | Range | 6.72 – 25.21 |
|  | Median | 14.62 |
|  | Mean ± SD | 14.74 ± 3.31 |
|  | IQR | 4.78 |
| V70 Gy (%) | Range | 3.72 – 18.02 |
|  | Median | 9.62 |
|  | Mean ± SD | 9.75 ± 2.57 |
|  | IQR | 3.50 |
| V76 Gy (%) | Range | 1.29 – 10.99 |
|  | Median | 5.14 |
|  | Mean ± SD | 5.35 ± 1.83 |
|  | IQR | 2.52 |
| V80 Gy (cm^3^) | Range | 0.00 ­– 10.14 |
|  | Median | 0.02 |
|  | Mean ± SD | 0.53 ± 1.52 |
|  | IQR | 0.28 |

*Abbreviations*: V40 Gy (%), the volume of structure receiving a 40 Grey radiation dose, expressed as a percentage; V 80 Gy (cm^3^), the volume of the structure receiving an 80 Grey radiation dose, in cubic centimeters; SD, standard deviation; IQR, interquartile range

**Supplemental Table 2.** Comparison of results of EBRT studies in localized prostate cancer

| Reference | Modality | Total Dose (GyRBE) | 5-year BFFS | 5-year biochemical failure rate |
| --- | --- | --- | --- | --- |
| Bryant et. al. | Proton | 78 | 99% (LR) | 3.5% |
| Takagi et. al. | Proton | 74 | 99% (LR), 90% (overall) | 16.2% |
| Slater et. al. | Mixed Proton-Photon | 74 | 75% (overall) | - |
| Hamdy et. al. | Photon | 74 | - | 14% |
| Michalski et. al. | Photon | 79.2 | - | 31% |
| This study | Proton | 79.2 | 97% (LR), 97% (overall) | 7.8% |

*Abbreviations*: GyRBE, Grey (relative biological effectiveness); BFFS, biochemical failure-free survival; LR, low-risk
